# Neuroanatomical changes seen in MRI in patients with cerebral metastasized breast cancer after radiotherapy

**DOI:** 10.1177/03008916211031301

**Published:** 2021-07-13

**Authors:** Antonia Reibelt, Michael Mayinger, Kai J. Borm, Stephanie E. Combs, Marciana N. Duma

**Affiliations:** 1 Department of Radiation Oncology, Klinikum rechts der Isar, Technical University of Munich, Munich, Bayern, Germany; 2 Department of Radiation Oncology, University of Zurich, Zurich, Switzerland; 3 Deutsches Konsortium für Translationale Krebsforschung (DKTK)–Partner Site Munich, Munich, Germany; 4 Institute of Radiation Medicine (IRM), Department of Radiation Sciences (DRS), Helmholtz Zentrum München, Neuherberg, Germany; 5 Department of Radiation Oncology, University of Jena, Jena, Germany

**Keywords:** Neuroanatomical changes, cerebral metastasized breast cancer, brain radiotherapy, FreeSurfer

## Abstract

**Purpose:**

To quantify neuroanatomical changes using magnetic resonance imaging (MRI) in patients with cerebral metastasized breast cancer after brain radiotherapy (RT).

**Methods:**

Fifteen patients with breast cancer with brain metastases who underwent whole brain RT (WBR), radiosurgery (RS), and/or hypofractionated stereotactic treatment (STX) were examined at four time points (TPs). A total of 48 MRIs were available: prior to RT (TP1), 5–8 months after RT (TP2), 9–11 months after RT (TP3), and >20 months after RT (TP4). Using automatic segmentation, 25 subcortical structures were analyzed. Patients were split into three groups: STX (receiving STX and RS), RS (receiving RS only), and WBR (receiving WBR at least once). After testing for a normal distribution for all values using the Kolmogorov-Smirnov test, a two-sided paired *t* test was used to analyze volumetric changes. For those values that were not normally distributed, the nonparametric Mann-Whitney test was employed.

**Results:**

The left cerebellum white matter (*p* = 0.028), the right pallidum (*p* = 0.038), and the left thalamus (*p* = 0.039) significantly increased at TP2 compared to TP1. The third ventricle increased at all TPs (*p* = 0.034–0.046). The left choroid plexus increased at TP3 (*p* = 0.037) compared to TP1. The left lateral ventricle increased at TP3 (*p* = 0.012) and TP4 (*p* = 0.027). Total gray matter showed a trend of volume decline in STX and WBR groups.

**Conclusions:**

These findings indicate that alterations in the volume of subcortical structures may act as a sensitive parameter when evaluating neuroanatomical changes and brain atrophy due to radiotherapy. Differences observed for patients who received STX and WBR, but not those treated with RS, need to be validated further.

## Introduction

Metastatic brain cancer (MBC) is 10 times more common than primary brain cancer and affects 20% to 40% of all patients with cancer.^1–3^ For about 5% of patients with breast cancer, MBC is expected to emerge at some point during the course of the disease,^4–6^ resulting in a poor outcome with a 1-year survival rate of around 50%.^7^ Treatment for patients with MBC usually consists of a combination of systemic chemotherapy, neurosurgery, and/or radiotherapy, depending on resectability, general health, and number of brain metastases. Radiosurgery (RS) or hypofractionated stereotactic radiotherapy (STX) is usually performed instead of after neurosurgery, whereas patients with an increased number of or diffuse brain metastases or leptomeningeal cancer are treated with whole brain radiotherapy (WBR).

With WBR potentially causing atrophy of brain tissue,^8^ changes in neurocognitive functioning have been observed after radiotherapy.^9^ Long-term changes occurring in subcortical structures of the brain after RS or STX in patients with breast cancer are not fully explored.

The aim of this study was to analyze with magnetic resonance imaging (MRI) changes in subcortical structures of the brain of patients with cerebral metastasized breast cancer after radiotherapy.

## Methods

**Figure 1.**
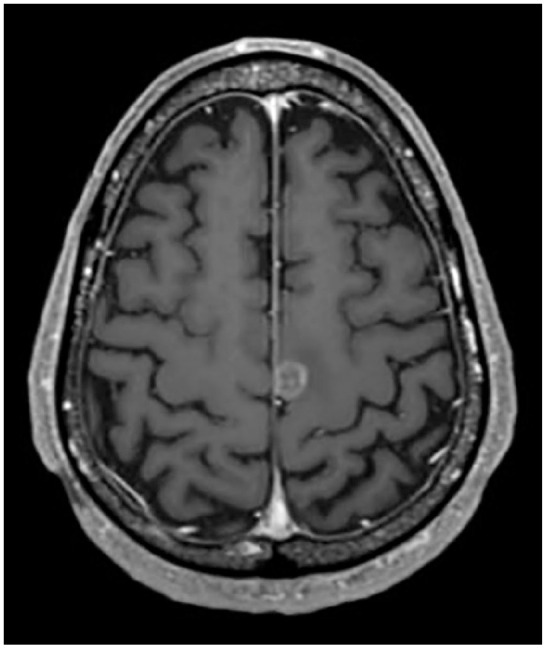
An exemplary magnetic resonance image of a patient treated for a single brain metastasis.

From our database, 135 women with breast cancer with secondary malignant neoplasms of the brain and/or cerebral meninges (ICD code C79.3)^10^ treated between 2011 and 2017 were selected. Out of this cohort, 15 patients had more than three MRIs in a T1-weighted MRI sequence and no significant edema, large resection cavities, or any kind of disrupted anatomical structures, thus allowing a final segmentation with the software FreeSurfer (Athinoula A. Martinos Center for Biomedical Imaging). An exemplary image to depict image quality is seen in Figure 1.

Table 1 shows the patients’ characteristics including age, luminal stage, Karnofsky Performance Score, chemotherapy, and symptoms. The median dose applied was 20 Gy for RS (range 15–20 Gy), 35 Gy for STX (30–35 Gy) in 5–6 fractions, and 30 Gy for WBR in 10 fractions. For this analysis, patients were divided into three groups according to the received radiotherapy procedure: group RS (only radiosurgery: four patients), group STX (radiosurgery as well as at least one STX: five patients), and group WBR (RS, STX, and WBR: six patients). For further analysis, two additional subgroups were defined: group oWBR (only whole brain radiation and radiosurgery, without any STX: three patients) and oSTX (only stereotactic radiotherapy and >1 or no radiosurgery: five patients). While patients in group oSTX received neurosurgery in the course of their disease, this was not the case for those in group oWBR. By creating these groups, it was analyzed to what extent neurosurgery acted as a confounding factor, when observing changing subcortical volumes. Any trends observed were illustrated using the R^2^ value. Out of all patients, 9 out of 15 patients received chemotherapy.

**Table 1.** Patient characteristics.

	Stereotactic radiotherapy	Radiosurgery	Whole brain radiotherapy
Number of patients	5	4	6
Age, y	47.4 ± 12.2	49.75 ± 16.4	43 ± 15.1
Luminal A	0/5	0/4	0/6
Luminal B	0/5	0/4	1/6
Luminal B/HER2+	3/5	4/4	1/6
HER2+	1/5	0/4	1/6
Triple-negative	1/5	0/4	2/6
Unknown	0/5	0/4	1/6
Time from breast cancer diagnosis to ICD 79.3, mo	47 ± 16.7	112.5 ± 51.7	47.8 ± 17.6
Median	54	102,5	48
Minimum	20	63	29
Maximum	60	182	71
Extracranial metastasis	3/5	3/4	4/6
Karnofsky Performance Score, %	80	80	70
Median	80	85	80
Minimum	70	70	60
Maximum	90	90	80
Systemic chemotherapy	4/5	1/4	4/6
Brain symptoms (headache, convulsion, tiredness, memory loss)	3/5	2/4	3/6

MRI was employed to examine potential differences in 45 subcortical brain structures at four different time points (TPs): TP1 (MRI prior to radiotherapy), TP2 (5–8 months after RT), TP3 (11–19 months after RT), and TP4 (>20 months after RT). Due to the course of disease, patients were scanned at slightly different TPs. We decided to group the time slots in order to provide a better overview for the reader. All patients chosen for this study had data acquired on a 3T MRI scanner (Magnetom Verio, Siemens Healthcare) with a 32-channel head coil array. T1-weighted 3D magnetization-prepared rapid gradient echo (MPRAGE) MRI scans in an axial orientation were used for data analysis. Imaging parameters were as follows: MPRAGE: repetition time 1800 ms, echo time 3.06 ms, field of view 256 mm, voxel size 1 × 1 × 1 mm^3^. In total, 48 MRIs were analyzed.

**Figure 2.**
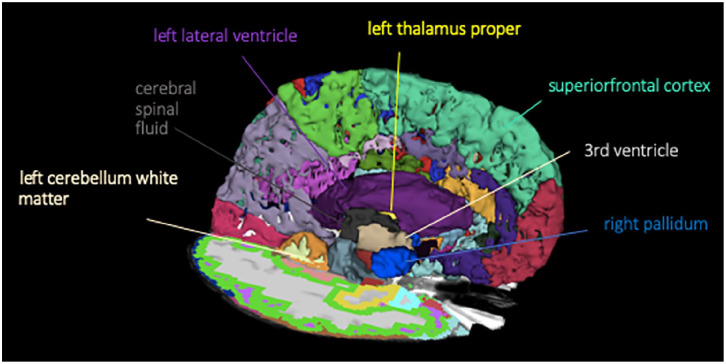
Subcortical surface segmentation showing all brain structures with statistically significant volume changes.

By creating an nhdr header for each subject, images were transformed from the DICOM to the NRRD file format. Using FreeSurfer version 5.3, cortical thickness analysis was carried out. Cortical reconstruction and volumetric segmentation were performed with the FreeSurfer image analysis suite (http://surfer.nmr.mgh.harvard.edu/). Further technical details of these procedures are described elsewhere.^11^ After aligning the images to a common atlas, which was initially created out of 50 healthy brains, the grayscale intensity was normalized and corrected for field inhomogeneity of the magnetic field. FreeSurfer analysis is separated into a cortical stream, which allows normalization and skull stripping of the brain, and a subcortical stream, allowing white matter segmentation. First, the cortical stream preprocesses the brain volume to a standardized 3D space, which is then used for the subcortical stream. The second stream uses the probabilistic atlas to assign different labels to subcortical structures. Voxels were labeled as gray matter (GM), white matter (WM), or cerebrospinal fluid (CSF). The GM (pial) and WM surfaces were created and the cortical surface was parcellated into discrete units based on both gyral and sulcal anatomy. Manual correction of the segmented units was performed to compensate for geometric inaccuracies arising from the occurrence of edema, state after surgery, or topologic defects. This was, however, only necessary for 3 out of 48 scans. An example for a subcortical segmentation can be seen in Figure 2. Alterations in volumes of the neuroanatomical structures were then analyzed between different subgroups, which were created in regard to their radiotherapy regimen. For the samples that were normally distributed, which was tested using the Kolmogorov-Smirnov test, statistical analysis was performed using 2-sided paired *t* tests for time intervals TP2, TP3, and TP4 compared with the initial MRI results for TP1. *p* Values ⩽ 0.05 were defined as statistically significant. For those values that were not normally distributed the nonparametric Mann-Whitney test was employed. Results were not adjusted for multiple testing. All data were analyzed using SPSS 25 (SPSS). Data are presented as median (range) and average with SD.

## Results

Follow-up MRI was available for 14 patients at TP2, 13 patients at TP3, and 6 patients at TP4. For the patients in group STX or RS, 5 out of 9 patients underwent radiotherapy for lesions located solely on the left hemisphere, whereas 1 underwent only right-sided radiotherapy. Three out of 9 patients underwent radiotherapy on both sides of the brain.

**Figure 3.**
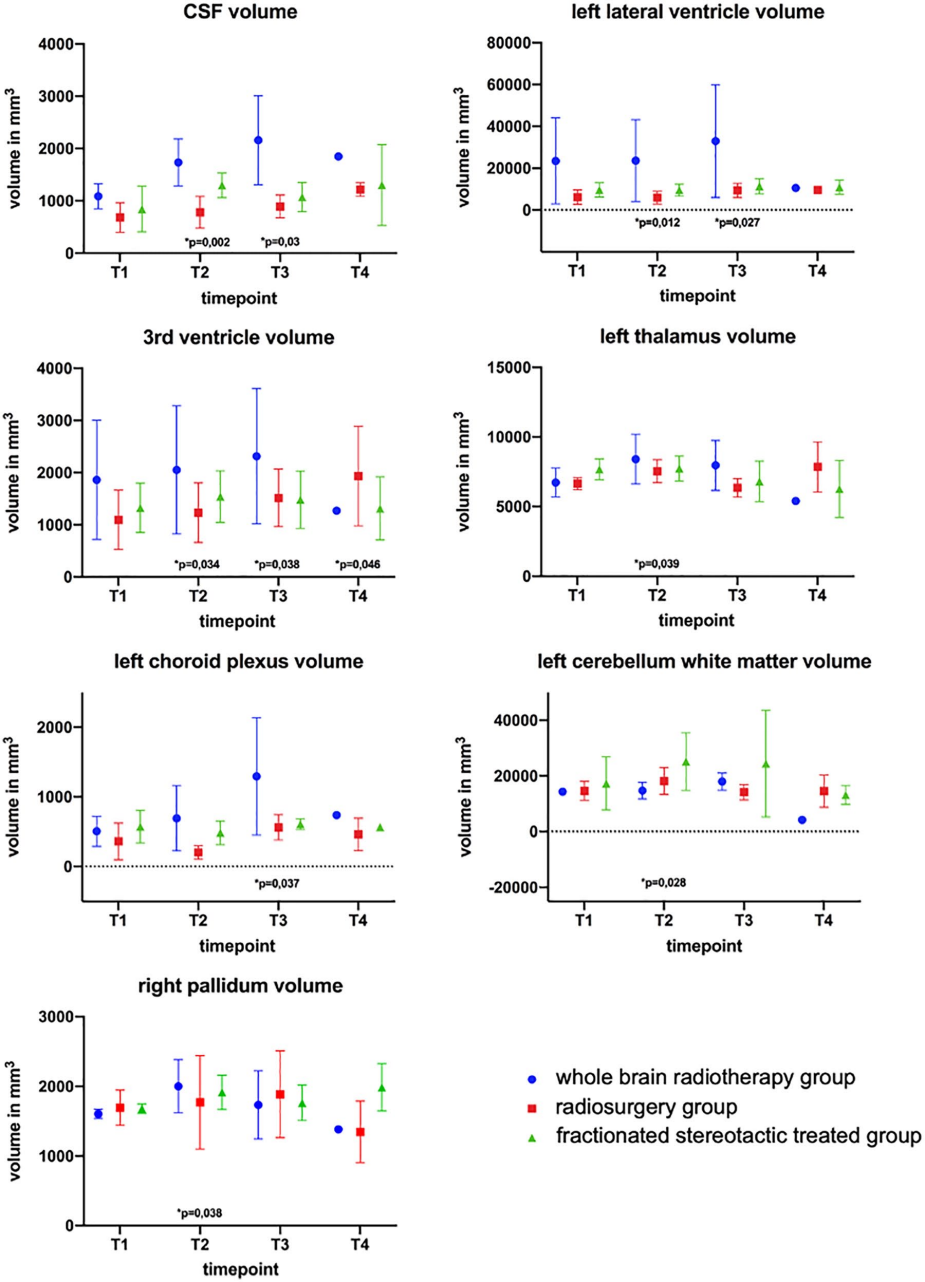
Subcortical brain structures with statistically significant volume differences with respect to TP1.

Table 2 displays the different radiotherapy modes that were performed for each patient at each TP. All subcortical brain structures presenting a statistically significant difference in volume are shown in Figure 3.

**Table 2.** Radiotherapy modes performed for each patient at the four different time points (TPs).

Patient	TP1	TP2	TP3	TP4
P_01	0	RS	STX	0
P_02	0	RS	0	RS
P_03	0	RS and STX	0	—
P_04	0	STX	2× WBRT	—
P_05	0	RS and WBRT	0	—
P_06	0	STX	WBRT	—
P_07	0	RS	0	—
P_08	0	STX	0	STX and WBRT
P_09	0	RS	0	2× RS
P_10	0	—	2× WBRT	0
P_11	0	STX	4× STX	STX
P_12	0	STX	—	—
P_13	0	RS	—	—
P_14	0	STX	0	—
P_15	0	WBRT	RS	—

—: No magnetic resonance imaging; 0: no radiotherapy; RS: radiosurgery; STX: stereotactic radiotherapy; WBRT: whole brain radiotherapy.

When comparing the subcortical brain structures of all 14 patients at TP2 to their initial MRI, the right pallidum (*p* = 0.038) and the left thalamus proper (*p* = 0.039) showed a significant increase in volume. No statistically significant volume changes in total WM volume were observed. The third ventricle also displayed an increased volume at all stages: TP2 (*p* = 0.034; 12%), TP3 (*p* = 0.038; 22%), and TP4 (*p* = 0.046; 56%). Furthermore, CSF increased in volume at TP2 (*p* = 0.002) and the left choroid plexus (CP) showed an increased size at TP3 (*p* = 0.0295), after employing the nonparametric Mann-Whitney Test. The left lateral ventricle showed an increase in volume at TP4 (*p* = 0.027).

**Figure 4.**
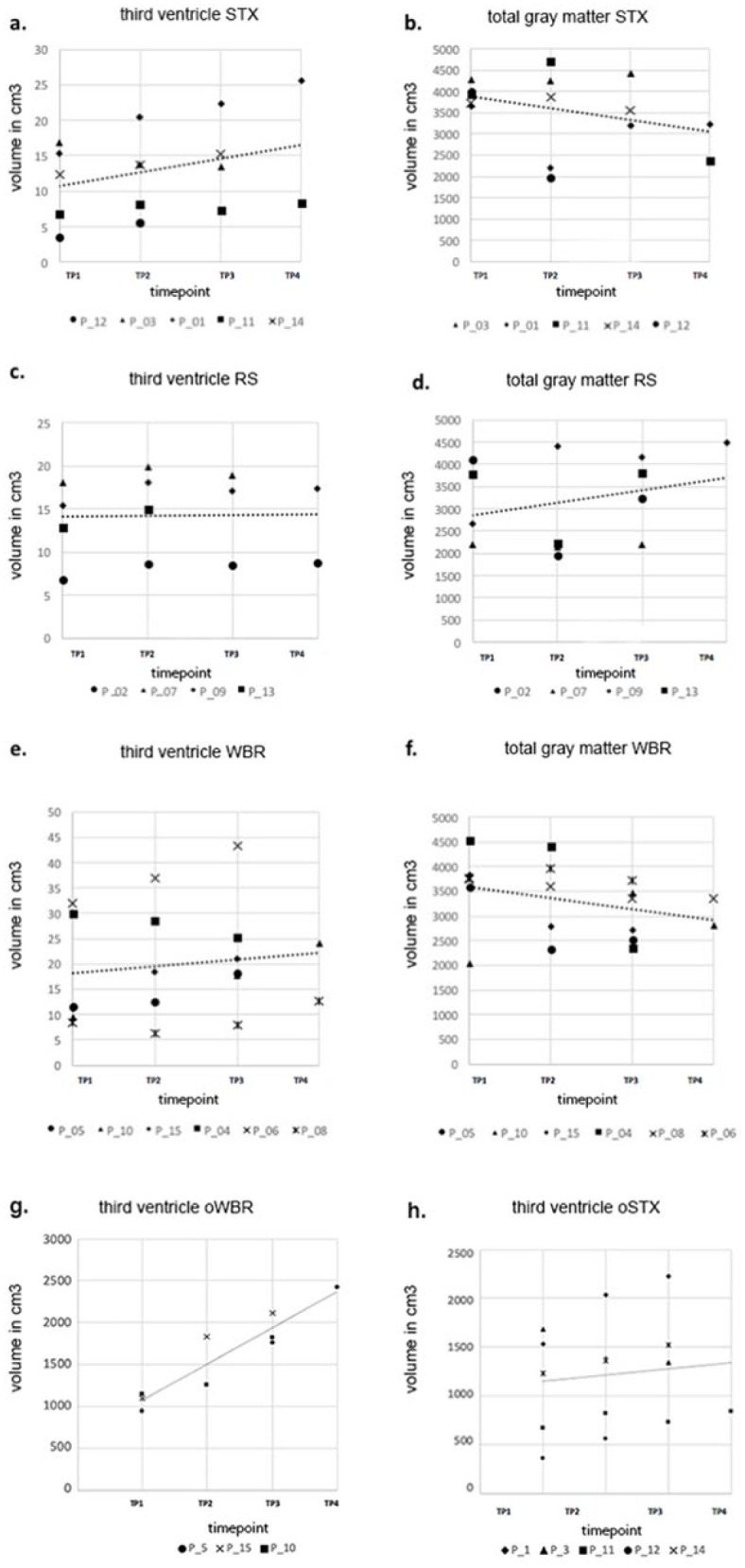
Volumetric changes of the third ventricle and gray matter (in cm^3^). The scatterplots depict third ventricle and gray matter volume for each patient. [a] and [b] show volumes of patients in group stereotactic radiotherapy (STX), [c] and [d] for group radiosurgery (RS), [e] and [f] for group whole brain radiotherapy (WBR), and [g] and [h] for groups oWBR (patients only treated with WBR) and oSTX (patients only treated with STX).

Although third ventricle volume changes seemed relevant to all groups, these changes were not equally pronounced among the specific subgroups. To analyze the different effects of different modes of radiotherapy, Figure 4 shows a clear representation of volumetric changes in the third ventricle among these groups. When considering all patients, the average increase in volume of the third ventricle was strongest for group STX and can be quantified by evaluating the linear trend for all time intervals after splitting patients into their respective groups (STX, RS, WBR). The coefficients of determination for the third ventricle volumes were R^2^ = 0.33 for group STX, R^2^ = 0.13 for group WBR and R^2^ = 0.02 for group RS.

No statistically significant volume changes in GM were observed. Nevertheless, a trend of volumetric decline can be seen for group STX (R^2^ = −0.35) and group WBR (R^2^ = −0.32) (Figure 4).

To evaluate whether ventricular enlargement was predominantly caused by radiotherapy or by a decrease of the surgery cavity, group WBR was analyzed in comparison with groups STX and RS regarding the enlargement of the third ventricle over time. The third ventricle enlargement was more pronounced in group oWBR, with a determination coefficient of R^2^ = 0.86759, compared to those in group oSTX (R^2^ = 0.01169). However, no statistically significant difference in ventricle dilation between those groups was observed.

## Discussion

With automatic segmentation being a relatively new approach in the analysis of subcortical structures, this is the first study to detect morphologic changes of cerebral metastasized breast cancer after radiotherapy over time. Any changes observed among the different groups will be discussed in regard to their location, as knowledge of these changes in volumes of various subcortical structures might alter treatment concepts, thus reducing side effects.

### Volumetric changes of the CP

The left CP, which is situated inside the ventricles and produces CSF, showed an increase at stage TP3 compared to TP1. Studies on patients with Alzheimer disease have shown that the CP plays a central role in blood–brain communication and changes in its cells are associated with neurodegenerative diseases.^12,13^ CP cells contain numerous transporters and are believed to actively secrete molecules that spread inflammatory responses in the brain.^12^ This pathway is activated by inflammation of the surrounding area and works via a secretion of CP epithelial derived extracellular vesicles into the CSF.^14^ Since a calcification and thickening of the basement membrane has also been observed in this process,^15^ the increase in size of the CP in our cohort might hint at its role in neuroinflammatory changes occurring after irradiation.

### Volumetric changes of the third ventricle

The increase in volume of the third ventricle at all stages may occur due to a decrease in overall cerebral volume. The present findings indicate a gray matter volumetric decline proportionate to the third ventricle dilation. The third ventricle is the smallest compartment of the ventricular system contoured in our study and might thus reflect even the smallest changes due to cerebral atrophy. A previous study has reported that WBR can cause cerebral atrophy as well as ventricle volume dilation of 20%–21%.^16^ As seen in Figure 4, the strongest decrease in GM volume was seen for group STX across all TPs, with the strongest increase in the volume of the third ventricle also being observed for group STX. It has to be noted that the R^2^ values for third ventricle volume changes are low. This may be due to the fact that no direct correlation between the mode of radiotherapy and volume changes has been found. Nonetheless, the observed trend should be noted and taken into account when evaluating these findings.

In order to test whether the observed increased volume of the third ventricle might be due to an increased intracranial space after neurosurgery, we compared the oSTX to the oWBR group. As the third ventricle enlargement was, however, more pronounced in oWBR, surgery does not seem to play a crucial part. According to a recent study, the resection cavity initially decreases on average by 23.4% (±41.5%). As time proceeds, the extent of cavity shrinking decreases continuously,^17^ with no statistically significant cavity size changes being observed 33 days after resection.^18^ This indicates that volume changes of the resection cavity may play a vital role right after surgery, but this effect can no longer be observed as time proceeds. In our cohort, changes in volume were analyzed up to 20 months after initial MRI. Hence, the effect of shrinking surgery cavities on the third ventricle at TP4 may be negligible and other pathogenic pathways have to be considered for persisting volume changes.

### Volumetric changes of the GM

Although this study did not observe significant volume changes of the GM, it did show a trend of GM decline for WBR and STX, which was indirectly proportionate to third ventricle dilation. Previous examinations have reported radiation-induced GM damage in patients receiving intensity-modulated radiotherapy.^19^ As radiotherapy-induced neurotoxicity affecting the GM may cause a decline in its volume, the increased intracranial space could in turn lead to an increase in third ventricle and CSF volume. A decrease in GM volume might entail negative consequences, as it has been linked to neurocognitive impairments and memory loss in patients with Alzheimer disease^20–22^ and long-term cognitive disability.^23^ Although not significant, an increase in GM volume was observed for the RS group. This is in line with a study by Brown et al.^24^ indicating that RS is likely to induce less neurocognitive impairment compared to WBR. Furthermore, literature has shown RS causes less decline in learning and memory function. A study by Chang et al.^25^ showed that there “was a high probability (96%) that patients randomly assigned to receive SRS plus WBRT were significantly more likely to show a decline in learning and memory function (mean posterior probability of decline 52%) at 4 months than patients assigned to receive SRS alone (mean posterior probability of decline 24%).” Besides this, alternative factors such as a more specific inflammation reaction in this group should be taken into account. Another study by Petr et al.^26^ has shown that “tissue volume decrease depends on radiation dose delivered to the healthy hemisphere and differs between treatment modalities.” Similar studies investigating the correlation of volumetric changes of the cortical and subcortical structures in correspondence to respective radiation doses should be conducted to allow for a thorough evaluation of the advantages and disadvantages of the present radiotherapy modalities in this context.

### Periventricular volumetric changes

The right pallidum and left thalamus showed an increased volume at TP2. The pallidum is part of the extrapyramidal motor system, while the thalamus functions to relay sensory and motor signals to the cerebral cortex and regulates sleep, alertness, and consciousness. As seen in multiple sclerosis (MS), periventricular structures are very sensitive to demyelination.^27^ Initial swelling after radiotherapy, as observed in the present study, could be an early indicator for future demyelination of those structures. Studies have shown that WM atrophy can be observed after irradiation^28^ and might be caused by an occurring demyelination, microvascular occlusion, or blood–barrier breakdown.^29^ Recent research on patients with MS has shown that WM demyelination and GM atrophy are related.^30^ Although rare cases of “acute demyelination following radiotherapy after glioma”^31^ have been reported, no sufficient research is available evaluating a possible comparison between pathogenic pathways of WM demyelination due to inflammation (i.e. caused by MS and due to radiotherapy). Nevertheless, this might be an interesting field to look into when aiming to explain the neuroanatomical changes occurring in the brains of patients with breast cancer during and after radiotherapy.

### Volumetric changes of the cerebellum

While we observed no significant alterations in total WM volume, the left cerebellum WM showed an increase in volume at TP2 (+24%). This might be explained by a subacute (directly after radiotherapy) inflammation and swelling of WM tissue, caused by subacute irradiation toxicity.^32^ Yamamoto et al.^33^ reported that out of 167 patients, 17 patients experienced delayed radiation complications 24 to 121 months after radiosurgery. At TP3, the left cerebellum WM volume increase dropped to +9% compared to TP1. At TP4, a decrease in left cerebellum WM volume was observed (–20%). Changes observed at TP4 may be due to delayed radiation complications as observed by Yamamoto et al.^33^ Volumetric changes at TP3 and TP4 were not statistically significant. These fluctuations might once more be considered as a consequence of a shrinking of the surgery cavities, leading to changes in WM volume dynamics. However, this effect should be rather small as only 2 out of 15 patients received neurosurgery on the cerebellum, with one patient receiving neurosurgery on both sides and the other only on the left side of the cerebellum, while all other patients received supratentorial surgery. The observed effect might at first only be seen in the left cerebellum WM and not the total cerebral WM, as this is a smaller subunit and our cohort received more irradiation of the left hemisphere. As WM swelling may in the long term result in white matter demyelination, medication prescribed for patients with MS to prevent white matter demyelination, such as corticosteroids, might reduce radiotherapy-induced neurotoxicity.

### Laterality of changes

In order to detect even minor changes in volume and be able to locate those changes precisely, we decided to subdivide neuroanatomical structures as much as FreeSurfer allowed us to, including left and right. Changes in bilateral subcortical structures were mostly observed in the left hemisphere (in the left thalamus, in the left CP, and in the left lateral ventricle). This might be caused by more patients in the STX and RS groups receiving radiation to the left hemisphere.

### Limitations

Partial volume effects for small structures cannot be excluded, even after manual correction. Another limitation of this study is represented by the small number of included patients, which has to be emphasized when interpreting the results. Because patients with breast cancer who develop brain metastases or leptomeningeal cancer have devastating outcomes, it is difficult to acquire large cohorts of patients with long-term follow-up MRI scans. A further limitation of the present study is the patients’ individualized radiotherapy regimens and differing numbers of metastases. This led to inhomogeneous groups and increased statistical deviations. Nine out of 15 patients received chemotherapy, which makes it challenging to distinguish between neuroanatomical transformations occurring due to radiotherapy and changes resulting from systemic chemotherapy.^34^ A study by Menning et al.^35^ comparing patients with breast cancer receiving systemic treatment (doxorubicin and cyclophosphamide ± taxanes ± endocrine treatment) to those with no systemic treatment observed subtle detrimental effects of chemotherapy ± endocrine treatment by showing a larger decline in WM integrity in the superior longitudinal fasciculus and corticospinal tract. Multicentered prospective longitudinal studies of large patient cohorts are warranted to confirm the present results.

## Conclusion

Several neuroanatomical alterations were observed over the course of time. The present results indicate that the third ventricle may act as a sensitive parameter when evaluating subcortical changes and possible future brain atrophy due to radiotherapy. An increase of the CP, an important blood–brain barrier gate, hints at this structure playing an important role in neurodegenerative changes after radiotherapy. Differences in GM volume decline observed for patients who received STX and WBR, but not those treated with RS, need to be validated further in comparison to healthy control groups to better evaluate possible treatment-related side effects.
